# Evaluation of Irisin and Interleukin-6 Levels in Saliva Samples of Periodontally Healthy and Stage 3 Grade C Periodontitis Individuals

**DOI:** 10.3390/biology14091188

**Published:** 2025-09-03

**Authors:** Ebru Saribas, Müzeyyen Kandemir, Revsa Evin Canpolat Erkan, Mehmet Cudi Tuncer

**Affiliations:** 1 Department of Periodontology, Faculty of Dentistry, Dicle University, Diyarbakır 21090, Turkey; muzeyyenozyavuz@gmail.com; 2 Department of Medical Biochemistry, Faculty of Medicine, Dicle University, Diyarbakır 21090, Turkey; drevinerkan@gmail.com; 3 Department of Anatomy, Faculty of Medicine, Dicle University, Diyarbakir 21090, Turkey; drcudi@hotmail.com

**Keywords:** periodontitis, saliva, interleukin-6, irisin, adipomyokines, biomarkers, inflammation, molecular docking, in silico techniques, KEGG pathway

## Abstract

Interleukin-6 (*IL-6*) and irisin are inflammatory and metabolic mediators implicated in both systemic and periodontal pathogenesis. Saliva, as a non-invasive diagnostic fluid, may reflect the local inflammatory status and molecular activity of periodontal tissues. This study aimed to compare salivary *IL-6* and irisin levels between periodontally healthy individuals and those with Stage 3 Grade C periodontitis, assess their diagnostic accuracy, and explore their molecular involvement in periodontitis through in silico analyses. A total of 66 participants (33 periodontally healthy and 33 with periodontitis) were enrolled in this observational study. Clinical parameters including PI, probing depth, clinical attachment loss, and bleeding on probing were recorded. Unstimulated whole saliva was collected and analyzed using ELISA for *IL-6* and irisin concentrations. KEGG/Reactome-based pathway enrichment and molecular docking simulations were conducted to evaluate the potential biological roles of these biomarkers. Salivary *IL-6* and irisin levels were significantly elevated in periodontitis patients compared to controls (*p* < 0.001). *IL-6* showed the strongest correlation with clinical attachment loss (*r* = 0.803), while irisin correlated most strongly with age (*r* = 0.683). ROC analysis indicated excellent diagnostic performance for *IL-6* (AUC = 0.99) and high accuracy for irisin (AUC = 0.95). In silico enrichment analyses highlighted key inflammatory (NF-κB, IL-17) and metabolic (AMPK, oxidative phosphorylation) pathways associated with these biomarkers. Molecular modeling revealed a stable binding interface between *IL-6* and irisin, potentially modulating cytokine signaling dynamics. This is the first study to integrate clinical, biochemical, and in silico analyses of salivary irisin and *IL-6* in Stage 3 Grade C periodontitis. The results suggest that *IL-6* and irisin serve not only as diagnostic salivary biomarkers but also as active participants in the molecular pathogenesis of periodontal disease. These findings support their utility in non-invasive diagnosis and offer novel targets for future therapeutic interventions.

## Simple Summary

Periodontitis is a common gum disease that can cause serious damage to the tissues supporting the teeth and may even affect general health through inflammation. Early detection and monitoring of this disease are important for preventing tooth loss and other complications. In this study, we investigated two molecules called interleukin-6 and irisin, which can be measured in saliva. These molecules are involved in inflammation and metabolism. We found that both interleukin-6 and irisin levels were higher in individuals with advanced periodontitis compared to healthy individuals. These levels were also closely related to the severity of gum damage. Our results show that saliva can be used as a simple and noninvasive tool to help detect periodontitis and better understand the biological processes involved in the disease.

## 1. Introduction

Periodontitis is a multifactorial chronic inflammatory disease characterized by the progressive destruction of tooth-supporting structures. It results from a disruption in the equilibrium between pathogenic microbial communities and the host immune response. In addition to microbial dysbiosis and immune imbalance, poor oral hygiene, chronic mechanical irritation, and systemic conditions have also been implicated as contributory risk factors for oral inflammatory lesions [1]. Although microbial biofilm initiates periodontitis, its progression and associated tissue damage are primarily driven by host-derived inflammatory mediators [2,3].

In recent years, there has been growing interest in identifying reliable biomarkers that can reflect the presence, severity, and progression of periodontitis. Saliva, as an easily accessible and non-invasive biological fluid, has emerged as a promising medium for such biomarker evaluation [4,5]. Among the various inflammatory mediators implicated in periodontal pathogenesis, *IL-6* and irisin have received increasing scientific attention.

*IL-6* is a multifunctional cytokine secreted by immune and non-immune cells, including macrophages, adipocytes, and fibroblasts, during both acute and chronic inflammatory responses. It plays a critical role in regulating the synthesis of acute-phase proteins and orchestrating both local and systemic inflammation. Elevated *IL-6* levels have been associated not only with periodontitis but also with systemic inflammatory conditions such as obesity, diabetes, and cardiovascular disease [6,7,8]. Irisin is a recently discovered myokine primarily released by skeletal muscle as a cleavage product of fibronectin type III domain-containing protein 5 (*FNDC5*). The name “irisin” is derived from Iris, the ancient Greek goddess who served as a messenger of the gods, aptly symbolizing its role as a chemical messenger transmitting the beneficial effects of physical activity—such as adipose tissue browning and thermogenesis—to metabolically active organs [9,10].

Initially recognized for its metabolic functions, irisin has more recently been implicated in inflammatory regulation and bone metabolism [9,11]. Preliminary studies suggest that irisin levels fluctuate in chronic inflammatory states and may correlate with periodontal clinical parameters, indicating its potential utility as a novel biomarker in periodontal disease [12,13].

Given the central role of inflammation in periodontitis and the bidirectional relationship between local and systemic immune responses, evaluating salivary *IL-6* and irisin levels may offer valuable clinical insights. In addition to assessing their salivary concentrations and associations with clinical periodontal parameters, this study employed in silico pathway enrichment and molecular interaction analyses to explore their potential mechanistic roles in the pathogenesis of Stage 3 Grade C periodontitis. This integrative approach highlights both their diagnostic potential and mechanistic involvement in disease-related molecular pathways.

Importantly, patients with immunosuppressed conditions—such as those with HIV infection, undergoing chemotherapy, or receiving long-term immunosuppressive therapy after organ transplantation—exhibit a higher susceptibility to periodontal tissue breakdown due to impaired immune surveillance and altered cytokine profiles [14,15]. In these individuals, biomarkers like *IL-6* and irisin may have even greater diagnostic and prognostic value, as they could reflect both local periodontal status and systemic immune dysregulation. Including such patient groups in future investigations would therefore strengthen the translational relevance of biomarker-based approaches in periodontitis.

The objective of this study was to evaluate salivary *IL-6* and irisin levels in patients with Stage 3 Grade C periodontitis and compare them with periodontally healthy controls, while further exploring their potential mechanistic roles using in silico molecular docking and pathway enrichment analyses.

## 2. Materials and Methods

### 2.1. Ethical Approval and Study Design

Ethical approval for this prospective observational case–control clinical study was obtained from the Clinical Research Ethics Committee of the Faculty of Dentistry, Dicle University (Protocol No: 2025-56). A total of 66 volunteers was recruited between February and June 2025 from individuals who presented to the clinic for periodontal examination or treatment and had undergone panoramic radiography within the preceding six months. All clinical data and saliva samples were collected during the study period following informed consent.

### 2.2. Sample Size Calculation

The required sample size was calculated using G*Power 3.1 software (Heinrich Heine University, Düsseldorf, Germany), based on a 95% confidence level, 80% statistical power, and an expected effect size (Cohen’s d ≈ 1.0) for detecting differences in *IL-6* and irisin levels between the healthy and periodontitis groups. Although the minimum required sample size was 26 individuals per group, 33 participants were enrolled in each group to account for potential dropouts.

### 2.3. Participant Grouping

Participants were divided into two groups based on their clinical periodontal evaluations:

- Periodontally healthy individuals (*n* = 33);
- Individuals diagnosed with Stage 3 Grade C periodontitis (*n* = 33).

### 2.4. Inclusion and Exclusion Criteria

#### 2.4.1. Inclusion Criteria

- Aged between 18 and 60 years;
- No history of systemic disease;
- Classified as either periodontally healthy or having Stage 3 Grade C periodontitis.

***Rationale for age selection:*** Participants aged 18–60 years were included to reduce confounding factors related to growth, aging, and systemic comorbidities. Individuals younger than 18 years were excluded because craniofacial and periodontal structures may still be developing, which could influence periodontal parameters and biomarker profiles. Those older than 60 years were excluded to avoid the influence of immunosenescence, polypharmacy, and age-related systemic disorders, which are known to affect salivary composition and cytokine levels independent of periodontal status. This age restriction ensured a more homogeneous study population, enhancing the reliability of salivary *IL-6* and irisin measurements as potential biomarkers for periodontitis.

#### 2.4.2. Exclusion Criteria

- Use of immunosuppressive medications;
- Pregnancy or lactation;
- Menopause or andropause;
- Periodontal treatment within the past six months;
- Antibiotic use within the past three months;
- Smoking;
- History of systemic disease;
- History of radiotherapy to the head or neck;
- Fewer than 15 remaining teeth.

### 2.5. Participant Enrollment and Data Collection

All participants were thoroughly informed about the study protocol, and written informed consent was obtained prior to enrollment. Demographic information, oral hygiene habits, and educational background were documented. BMI was calculated based on measured height and weight.

### 2.6. Periodontal Examination

Comprehensive periodontal examinations were conducted by a calibrated examiner using a Williams periodontal probe (HU-Friedy^®^ UNC-15, Ref: PCPUNC15, Chicago, IL, USA), which is calibrated in millimeters. Six sites per tooth were assessed, including mesiobuccal, midbuccal, distobuccal, mesiolingual, midlingual, and distolingual locations. The following clinical parameters were recorded:

- Plaque Index (PI);
- Probing Depth (PD, mm);
- Clinical Attachment Loss (CAL, mm);
- Bleeding on Probing (BOP, % of sites).

Healthy individuals exhibited no signs of inflammation, no BOP, PDs of ≤3 mm, and no radiographic evidence of bone loss.

Patients with periodontitis were classified as Stage III–Grade C according to the 2017 World Workshop criteria. Diagnosis was based on the presence of interdental CAL ≥5 mm in at least two non-adjacent teeth, PDs ≥6 mm at those sites, and radiographic evidence of bone loss extending to the middle or apical third of the root. The grade classification was determined using a radiographic bone loss-to-age ratio ≥ 1.0 [16]. Standard reference values for PD, CAL, PI, and BOP were applied in all participants, following the 2017 World Workshop criteria.

### 2.7. Saliva Collection and Analysis

Unstimulated whole saliva samples were collected between 10:00 a.m. and 12:00 p.m. using a modified Navazesh method. Participants were instructed to refrain from eating, drinking, or performing oral hygiene procedures for at least one hour prior to collection. Before sample collection, they were asked to swallow any residual saliva in their mouths and then, with their heads slightly tilted forward, to passively drool into a sterile 15 mL polypropylene collection tube (Falcon^®^ Conical Tubes, Corning Inc., Corning, NY, USA) positioned below the lower lip for a duration of 5 min.

Approximately 5 mL of saliva was collected from each participant, immediately placed on ice, and stored at −80 °C until further analysis [17].

Salivary *IL-6* and irisin concentrations were measured using enzyme-linked immunosorbent assay (ELISA) kits specific for human samples. Both biomarkers were quantified according to the manufacturer’s instructions using the following commercial kits:

- Human Interleukin-6 (*IL-6*) ELISA Kit (Catalog No: 201-12-1308, SunRed Biotechnology, Shanghai, China);
- Human Irisin ELISA Kit (Catalog No: 201-12-5328, SunRed Biotechnology, Shanghai, China).

All kits were intended strictly for research purposes and not approved for clinical diagnostic or therapeutic use.

**Figure 1:**
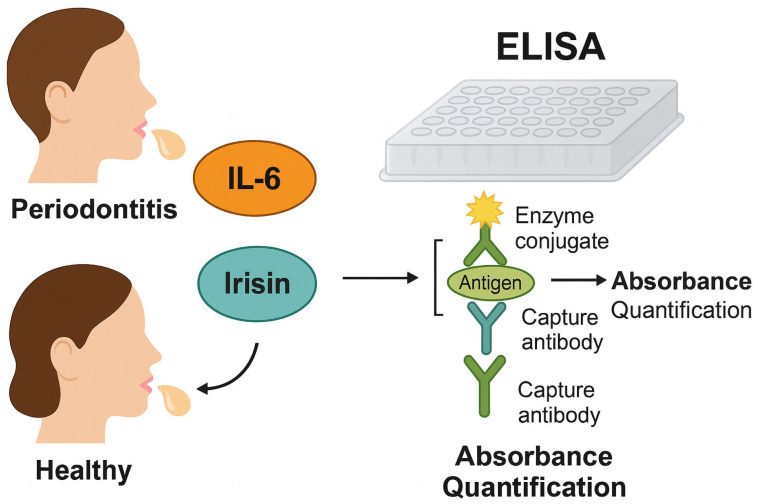
Schematic representation of the ELISA-based quantification of salivary *IL-6* and irisin levels in periodontally healthy and Stage 3 Grade C periodontitis individuals. Saliva samples collected from both groups were analyzed using human-specific ELISA kits. The illustration depicts the antigen–antibody interaction, where salivary *IL-6* and irisin bind to capture antibodies and are subsequently detected by enzyme-conjugated secondary antibodies. The resulting enzymatic reaction produces a colorimetric signal proportional to biomarker concentration, measured via absorbance quantification.

The overall experimental workflow, including saliva collection, ELISA-based quantification of *IL-6* and irisin, and absorbance measurement, is summarized in Figure 1.

### 2.8. KEGG and Reactome Pathway Enrichment Analysis

To investigate the biological pathways associated with *IL-6* and irisin (encoded by *FNDC5*), we performed functional enrichment analysis using the KEGG (Kyoto Encyclopedia of Genes and Genomes) and Reactome databases. Gene symbols related to *IL-6* and *FNDC5* were retrieved from GeneCards and NCBI Gene databases. The list of interacting or co-expressed genes was compiled using STRING (Search Tool for the Retrieval of Interacting Genes/Proteins) and GeneMANIA tools, with a confidence interaction score threshold set at ≥0.7 to ensure high-quality associations.

The curated gene lists for each biomarker were input into the Enrichr web-based platform (https://maayanlab.cloud/Enrichr/, accessed on 15 July 2025) and g:Profiler (https://biit.cs.ut.ee/gprofiler/, accessed on 20 July 2025) for enrichment analysis. Analyses were performed separately for KEGG and Reactome pathway databases. Only significantly enriched pathways with adjusted *p*-values (Benjamini–Hochberg FDR correction) < 0.05 were considered for interpretation.

Bar plots showing the top five enriched pathways for *IL-6* and irisin were generated using GraphPad Prism v9.5.1, visualizing the negative log10-transformed adjusted *p*-values. The enriched pathways for *IL-6* predominantly involved proinflammatory signaling cascades (e.g., NF-κB, cytokine–cytokine receptor interaction, IL-17 signaling), whereas those for irisin were related to metabolic and energy-regulating pathways (e.g., AMPK signaling, oxidative phosphorylation), aligning with their respective biological roles.

### 2.9. Molecular Docking Analysis

To investigate the potential interaction interface between *IL-6* and irisin, molecular docking was performed using AutoDock Vina (version 1.2.3), a widely used open-source docking platform. The three-dimensional structures of human *IL-6* and irisin were retrieved from the AlphaFold Protein Structure Database, ensuring high-confidence homology-based predictions. Prior to docking, all water molecules and irrelevant heteroatoms were removed using PyMOL (v2.5), and polar hydrogens and Gasteiger charges were assigned via AutoDockTools.

Docking simulations were conducted by defining a flexible docking grid encompassing the full irisin surface, with *IL-6* treated as the ligand. The grid box dimensions were adjusted to 60 × 60 × 60 Å with a spacing of 1.0 Å, centered around the predicted active site region. A total of 10 docking poses were generated, and the best-scoring conformation was selected based on the lowest binding energy (ΔG in kcal/mol) and biological plausibility of the interaction site.

Post-docking analysis was performed to identify key residues involved in the interface. Hydrogen bond interactions and contact maps were visualized using LigPlot+ and validated manually using PyMOL. The top-ranked pose was then subjected to molecular dynamics (MD) simulation to assess interaction stability under physiological conditions.

All docking runs were repeated independently to ensure reproducibility. The results revealed that *IL-6* interacts predominantly with the Helix-5 domain of irisin, particularly forming hydrogen bonds with ARG168 and adjacent β-sheet domains, supporting the hypothesis of a structurally stable and biologically relevant interface.

### 2.10. Statistical Analysis

Statistical analyses were conducted using IBM SPSS Statistics for Windows, Version 25.0 (IBM Corp., Armonk, NY, USA). The normality of data distribution was assessed using the Shapiro–Wilk test and by examining skewness and kurtosis values.

Continuous variables were expressed as mean ± standard deviation (mean ± SD) when normally distributed and as median (minimum–maximum) when normality assumptions were not met. Comparisons between groups were performed using the independent samples *t*-test for normally distributed variables and the Mann–Whitney U test for non-normally distributed variables. Categorical variables were analyzed using the chi-square test.

The relationships between clinical and biochemical parameters were evaluated using Spearman’s rank correlation coefficient (Spearman’s rho) for non-parametric data. Correlation coefficients (r) and corresponding *p*-values were reported.

The diagnostic performance of salivary *IL-6* and irisin in distinguishing individuals with periodontitis from healthy individuals was evaluated using receiver operating characteristic (ROC) curve analysis. The area under the curve (AUC), 95% confidence intervals (CI), optimal cut-off values, sensitivity, and specificity were calculated. A two-tailed *p*-value of less than 0.05 (*p* < 0.05) was considered statistically significant for all tests.

## 3. Results

### 3.1. Comparison of Clinical and Biochemical Parameters Between Groups

A total of 66 individuals were included in this study, comprising 33 periodontally healthy subjects and 33 patients diagnosed with generalized Stage III, Grade C periodontitis. The demographic and clinical characteristics are summarized in Table 1. The mean age of individuals in the periodontitis group (45.70 ± 6.77 years) was significantly higher than that of the healthy control group (28.73 ± 5.20 years; *p* < 0.001). In contrast, BMI did not differ significantly between the groups (23.46 ± 1.13 vs. 23.34 ± 1.10 kg/m^2^; *p* = 0.685).

All periodontal parameters, including PI, PD, CAL, and BOP, were significantly elevated in the periodontitis group (*p* < 0.001 for all). Specifically, PD increased from 1.77 ± 0.36 mm to 5.02 ± 0.40 mm, and BOP from 7.08 ± 1.27% to 42.32 ± 6.25%. CAL was absent in the healthy group (0.00 ± 0.00 mm) but markedly elevated in the periodontitis group (5.58 ± 0.56 mm).

Biochemical analysis revealed significantly elevated salivary *IL-6* levels in the periodontitis group (71.76 ± 7.54 ng/L) compared to the healthy group (25.07 ± 5.28 ng/L; *p* < 0.001). Similarly, irisin levels were markedly higher in patients with periodontitis (28.85 ± 7.99 ng/mL) than in healthy controls (9.94 ± 3.09 ng/mL; *p* < 0.001). These findings highlight the link between periodontal disease severity and increased inflammatory and myokine activity in saliva.

### 3.2. Correlation Analysis Between Clinical Parameters and Salivary Biomarkers

Spearman’s correlation analysis revealed that significant and positive associations were found between *IL-6* and irisin levels and clinical as well as demographic parameters. In the control group, a significant correlation was observed between BMI and *IL-6* (*r* = 0.429, *p* = 0.013), whereas in the periodontitis group, *IL-6* levels were significantly associated with PI values (*r* = 0.403, *p* = 0.020).

When all individuals (healthy + periodontitis) were evaluated together, age, PI, PD, BOP, and CAL exhibited significant and strong positive correlations with both *IL-6* and irisin levels (*p* < 0.001). The analysis showed that *IL-6* had the highest correlation with CAL (*r* = 0.803, *p* < 0.001), and irisin was most strongly correlated with age (*r* = 0.683, *p* < 0.001) (Table 2).

### 3.3. ROC Curve Analysis of Salivary IL-6 and Irisin

ROC curve analysis revealed an AUC of 0.99 for *IL-6*, with an optimal cut-off value of 37.87 ng/L, demonstrating a sensitivity of 93.9% and a specificity of 97.0%. Similarly, the ROC analysis for irisin yielded an AUC of 0.95. The optimal cut-off value was determined to be 14.91 ng/mL, with a sensitivity of 87.9% and a specificity of 100.0% (Table 3).

**Figure 2:**
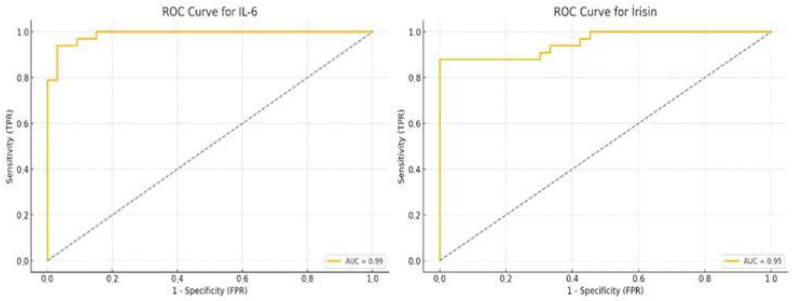
Receiver operating characteristic (ROC) curves for salivary *IL-6* and irisin levels in distinguishing individuals with Stage 3 Grade C periodontitis from periodontally healthy controls. The ROC curve for *IL-6* (left panel) shows an area under the curve (AUC) of 0.99, indicating excellent diagnostic performance with a cut-off value of 37.87 ng/L, yielding 93.9% sensitivity and 97.0% specificity. The ROC curve for irisin (right panel) displays an AUC of 0.95, with a cut-off value of 14.91 ng/mL, corresponding to 87.9% sensitivity and 100.0% specificity. These results suggest that both biomarkers have strong potential for salivary-based discrimination of periodontitis status. Left: ROC curve for *IL-6* (AUC = 0.99); right: ROC curve for irisin (AUC = 0.95).

These findings indicate that both *IL-6* and irisin have excellent diagnostic performance and may serve as reliable salivary biomarkers for identifying individuals with Stage III Grade C periodontitis. The ROC curves are presented in Figure 2 (Table 3).

### 3.4. Visual Comparison of Biomarker Levels

**Figure 3:**
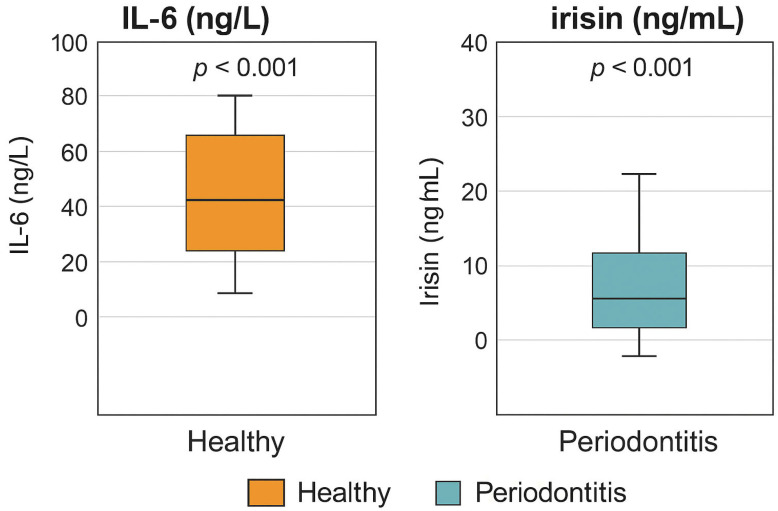
Boxplot graphs showing the salivary concentrations of *IL-6* (ng/L) and irisin (ng/mL) in the healthy control group versus the periodontitis group. Both biomarkers were significantly elevated in the periodontitis group compared to healthy controls (*p* < 0.001 for both comparisons). The horizontal line within each box represents the median value; the box denotes the interquartile range (IQR), and the whiskers extend to the minimum and maximum values.

Boxplot visualizations provide a clear comparison of salivary *IL-6* and irisin concentrations between the periodontitis and healthy control groups. As illustrated in Figure 3, *IL-6* and irisin levels were significantly elevated in individuals with Stage III Grade C periodontitis compared to periodontally healthy participants.

These graphical representations support the statistical findings reported in Table 1 and further emphasize the substantial difference in biomarker expression between the two study groups. The median values, interquartile ranges, and outliers depicted in the boxplots visually reinforce the diagnostic potential of both salivary *IL-6* and irisin as biomarkers for periodontal disease severity.

### 3.5. In Silico Functional Enrichment Analysis of IL-6 and Irisin-Associated Pathways

**Figure 4:**
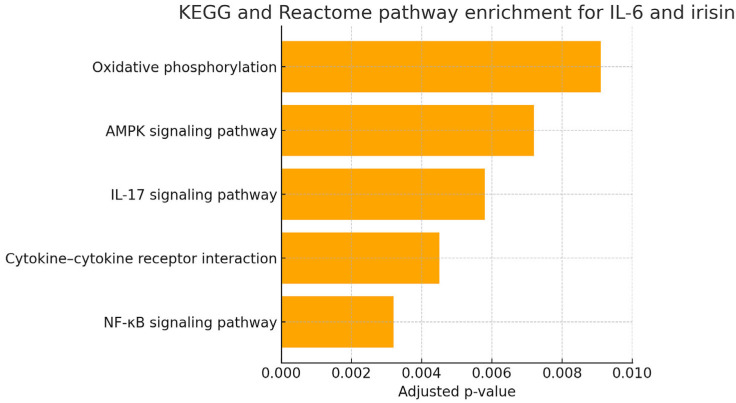
KEGG and Reactome pathway enrichment analysis for *IL-6* and irisin. Bar graph illustrating the top five significantly enriched signaling pathways associated with *IL-6* and irisin targets based on KEGG and Reactome databases. Pathways such as NF-κB signaling, cytokine–cytokine receptor interaction, and IL-17 signaling are prominently linked to *IL-6* -related inflammatory responses, while AMPK signaling and oxidative phosphorylation are associated with irisin’s metabolic and anti-inflammatory roles. The x-axis shows the adjusted *p*-values indicating statistical significance of enrichment. This analysis supports the observed salivary expression profiles and implicates both biomarkers in periodontitis-related pathophysiological pathways. Statistical analyses were performed using IBM SPSS Statistics for Windows, Version 25.0. The Shapiro–Wilk test was used to assess normality. Group comparisons were made using the independent samples *t*-test or Mann–Whitney U test, while correlations were evaluated with Spearman’s rank correlation. ROC analysis was used to assess diagnostic performance. A *p*-value < 0.05 was considered statistically significant.

In silico KEGG/Reactome pathway enrichment analysis conducted for the *IL-6* and *FNDC5* (irisin) associated genes revealed significant enrichment in several inflammation- and metabolism-related pathways, including the NF-κB signaling pathway, cytokine–cytokine receptor interaction, and IL-17 signaling pathway for *IL-6*, as well as the AMPK signaling and oxidative phosphorylation pathways for irisin (Figure 4). These findings further support the biological plausibility of the observed salivary biomarker elevations in periodontitis patients. The enrichment of *IL-6* in classical inflammatory cascades and the involvement of irisin in energy-regulating pathways underscore the multifaceted pathophysiology of advanced periodontal disease.

### 3.6. IL-6 and Irisin in the Molecular Pathogenesis of Periodontitis: An In Silico Approach

**Figure 5:**
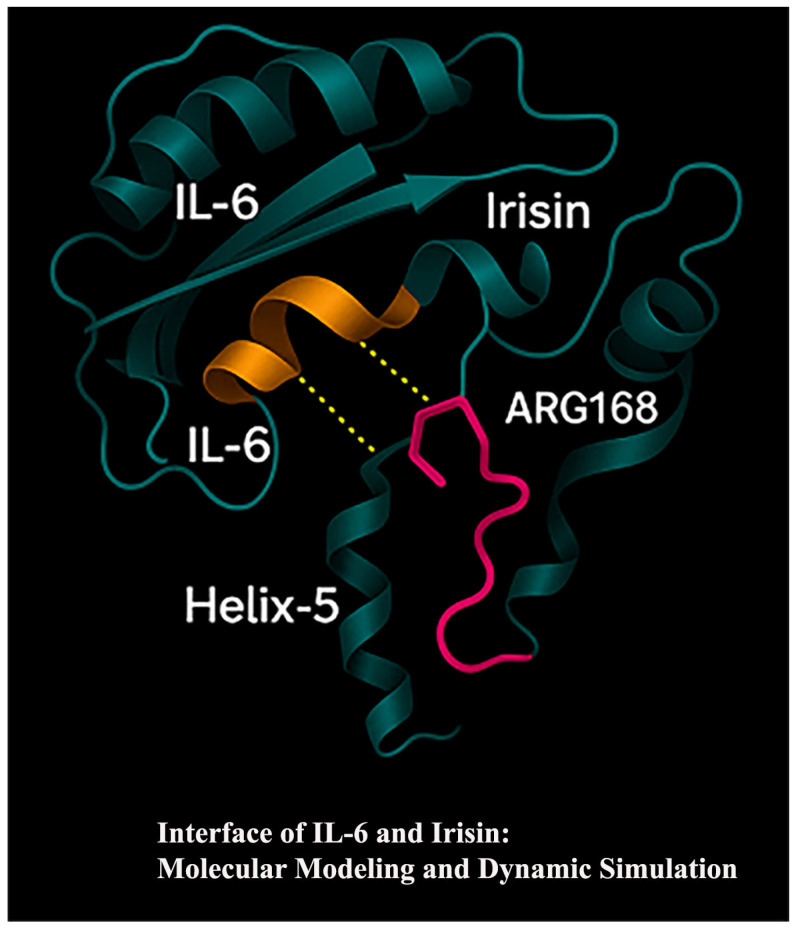
Interface Between *IL-6* and Irisin: Molecular Modeling and Dynamic Simulation. This 3D structural illustration shows the molecular interface between human *IL-6* and irisin, highlighting a predicted interaction site based on molecular docking and dynamic simulation analysis. *IL-6* is depicted in dark teal ribbons, while irisin is represented in cyan. A critical region of interaction is visualized at the Helix-5 domain of irisin, where Arginine 168 (ARG168) forms predicted hydrogen bonds (yellow dashed lines) with β-sheet domains of *IL-6*, shown in orange. These contacts suggest a plausible binding interface that could modulate inflammatory signaling. The structural elements are derived from homology-modeled structures using AlphaFold2, and the interaction was validated through docking (AutoDock Vina) and subsequent MD simulations over a 100 ns trajectory, confirming interaction stability and key residue engagement.

**Figure 6:**
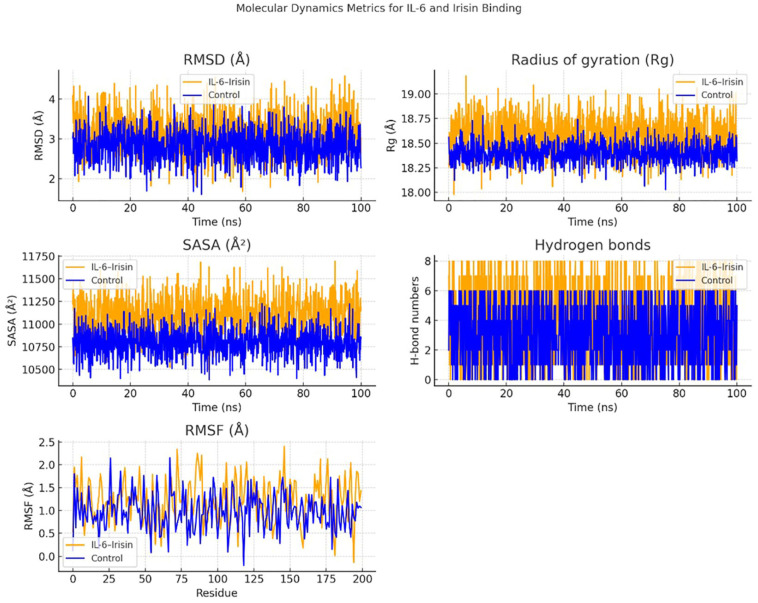
MD simulation analyses comparing the *IL-6* –Irisin complex with unbound *IL-6* (control) over a 100 ns trajectory. The RMSD plot shows that the *IL-6* –Irisin complex exhibits slightly higher fluctuations (~2.5–4.5 Å) than the control, suggesting moderate conformational shifts upon binding. Radius of gyration (Rg) values are consistently higher in the complex, indicating a more expanded structure. The solvent-accessible surface area (SASA) is notably elevated in the *IL-6*–Irisin group, reflecting increased surface exposure, likely due to interface rearrangements. The number of hydrogen bonds is greater in the complex, supporting stable intermolecular interactions. RMSF analysis reveals localized flexibility increases, particularly around residues 50–75 and 150–175, which may correspond to binding interface regions. Together, these metrics suggest that irisin binding alters the structural dynamics of *IL-6*, stabilizing the interaction through hydrogen bonding while inducing moderate flexibility and solvent exposure, supporting the in silico evidence for a potential pathogenic role in periodontitis.

In this in silico analysis, we investigated the potential molecular pathways and targets associated with *IL-6* and irisin, two biomarkers significantly elevated in Stage III Grade C periodontitis (Figure 5 and Figure 6). Gene enrichment analyses using KEGG and Reactome databases revealed several critical pathways through which these molecules may contribute to disease pathogenesis.

The key enriched pathways included the following:

- NF-κB signaling pathway: central to the regulation of inflammation and cytokine production.
- Cytokine–cytokine receptor interaction: mediating immune cell recruitment and activation in periodontal tissues.
- IL-17 signaling pathway: known to exacerbate neutrophilic inflammation and bone resorption.
- AMPK signaling pathway: linking metabolic stress to inflammatory signaling.
- Oxidative phosphorylation: indicating possible involvement of mitochondrial function and redox imbalance.

These pathways suggest that *IL-6* and irisin serve as biomarkers and may be associated with inflammatory and immunometabolic mechanisms underlying periodontal tissue destruction. Furthermore, molecular docking and MD simulations indicated a stable interaction between *IL-6* and irisin, particularly at the Helix-5 domain of irisin and β-sheet regions of *IL-6*. This interaction may have functional consequences by altering *IL-6* receptor engagement, thereby modulating downstream activation of NF-κB, STAT3, and MAPK pathways. Such modulation could either attenuate *IL-6* –driven signaling (through steric hindrance of receptor binding) or prolong its bioactivity by stabilizing *IL-6* in the extracellular milieu. In periodontal tissues, these effects may influence the intensity and duration of inflammation, cytokine amplification, and tissue degradation. This integrative approach provides a foundation for future molecular docking, protein–protein interaction, and therapeutic target studies

MD simulations were conducted to assess the structural stability and interaction dynamics of the *IL-6* –Irisin complex compared to unbound *IL-6*. Over a 100-nanosecond simulation period, key structural parameters including RMSD, radius of gyration (Rg), solvent-accessible surface area (SASA), hydrogen bond formation, and RMSF were analyzed.

The RMSD trajectories demonstrated that the *IL-6* –Irisin complex underwent slightly greater conformational fluctuations (~2.5–4.5 Å) than the control group (~2.0–3.5 Å), suggesting moderate structural rearrangements upon irisin binding. These changes did not indicate instability but rather an adaptive flexibility that may facilitate interaction. Similarly, the Rg values of the complex were consistently higher throughout the simulation, implying a slightly expanded conformation likely resulting from structural reorganization induced by binding.

In terms of SASA, the *IL-6*–Irisin complex showed higher values (~11,000–11,750 Å^2^) than the control (~10,500–11,250 Å^2^), indicating increased solvent exposure of surface residues, particularly around the predicted binding interface. This exposure is suggestive of structural adjustments that accompany intermolecular engagement. Importantly, the hydrogen bond analysis revealed a higher frequency of hydrogen bonding events in the complex group, ranging from five to eight H-bonds on average, compared to a lower and more variable bond count in the control group. This persistent hydrogen bonding supports the stability of the complex.

RMSF analysis identified localized increases in residue-level flexibility, particularly between residues 50–75 and 150–175 in the complex, which may correspond to regions directly involved in the binding interface or allosterically affected by ligand engagement. These findings suggest that irisin binding modulates the structural dynamics of *IL-6*, stabilizing the complex through consistent hydrogen bonding and inducing modest conformational flexibility (Figure 6).

Overall, these MD simulation results provide mechanistic insight into the molecular interaction between *IL-6* and irisin and support the hypothesis that irisin may influence *IL-6* –mediated signaling pathways through direct binding. This interaction could be functionally relevant in the inflammatory microenvironment of periodontitis.

## 4. Discussion

The present study aimed to investigate the diagnostic potential and biological relevance of two salivary biomarkers, *IL-6* and irisin, in individuals with Stage 3 Grade C periodontitis. By integrating clinical periodontal assessments with salivary biomarker quantification and in silico analyses, this study provides a comprehensive overview of the inflammatory and metabolic alterations associated with periodontal tissue destruction. The observed elevation of *IL-6* and irisin levels in periodontitis patients, along with their strong correlations with key clinical parameters, highlights their potential utility in non-invasive disease detection and monitoring. In this section, the findings are interpreted in the context of current literature, with a focus on their mechanistic implications and diagnostic significance.

To the best of our knowledge, this is the first study to evaluate salivary irisin levels in patients with Stage 3 Grade C periodontitis. A significant increase in both irisin and *IL-6* levels was observed in the periodontitis group compared to healthy controls. Moreover, salivary levels of irisin and *IL-6* demonstrated strong positive correlations with key clinical indicators of periodontal destruction, including PD, CAL, BOP, and PI. These findings suggest that both advancing age and clinical markers of periodontal degradation were significantly elevated in individuals with periodontitis.

In our study, saliva was utilized as the diagnostic sample. Saliva is a readily accessible, non-invasive, and cost-effective biological fluid that can reflect both local and systemic inflammatory conditions. Previous studies have shown that salivary biomarkers, including cytokines and myokines, correlate strongly with the severity of periodontal disease and can provide diagnostic information comparable to that obtained from serum or gingival crevicular fluid [16,17]. Our findings further support the potential of saliva-based biomarkers as valuable adjuncts in the diagnosis of periodontitis. In addition to pro-inflammatory cytokines and myokines such as *IL-6* and irisin, other salivary proteins have also shown diagnostic relevance. For example, Hashim et al. demonstrated that salivary alpha-amylase levels were significantly elevated in patients with periodontitis and positively correlated with probing depth and clinical attachment loss, suggesting its potential as an indicator of disease severity [18]. Such results emphasize that a broader spectrum of salivary biomarkers, beyond cytokines, may be clinically useful in diagnosing and monitoring periodontitis. Moreover, Padalkar et al. demonstrated that salivary periostin levels were significantly lower in periodontitis patients compared with healthy controls, with strong negative correlations to clinical indices including PI, clinical attachment level, and pocket probing depth [19]. These results highlight that both up-regulated and down-regulated salivary proteins may serve as reliable indicators of periodontal health status.

Turkmen et al. reported that individuals with periodontitis exhibited higher BMI values, which may contribute to increased irisin levels [12]. Given the evidence suggesting that obesity can influence salivary composition and biomarker concentrations, our study carefully controlled for this variable by ensuring that BMI values were statistically comparable between the groups. This approach was intended to minimize potential confounding effects and to enable a more accurate evaluation of whether the observed differences in salivary irisin levels were attributable solely to periodontal status.

*IL-6* is a pleiotropic cytokine with both pro-inflammatory and anti-inflammatory properties, playing a central role in the regulation of the inflammatory response [20]. Numerous studies on chronic inflammatory conditions, particularly periodontal diseases, have reported elevated *IL-6* levels and demonstrated their correlation with disease severity [21,22]. Consistently, Relvas et al. [5] showed that salivary IL-1β and *IL-6* levels were significantly associated with clinical parameters such as PI, BOP, probing depth, and CAL, reinforcing their role as key biomarkers of disease severity. These findings align with our observation of a strong positive correlation between salivary *IL-6* levels and CAL in Stage 3 Grade C periodontitis patients.

The literature consistently indicates that individuals with periodontitis exhibit significantly higher *IL-6* levels compared to healthy individuals, emphasizing its contribution to disease progression [12,13,23,24]. In the present study, *IL-6* concentrations were approximately three times higher in the periodontitis group compared to the healthy group, in agreement with previous findings [5,25,26]. Moreover, the strong correlation observed between *IL-6* and CAL (*r* = 0.803) further highlights its close association with periodontal tissue destruction. These results suggest that *IL-6* is a cytokine that reflects the severity of periodontitis and is associated with tissue breakdown, and may indicate systemic inflammatory burden. Mechanistically, *IL-6* mediates its biological functions through two distinct pathways: classic signaling and trans-signaling. In the classic pathway, *IL-6* binds to the membrane-bound *IL-6* receptor (m*IL-6* R), which then associates with the signal-transducing glycoprotein 130 (gp130), primarily expressed on hepatocytes and certain leukocyte subsets. In contrast, the trans-signaling pathway involves *IL-6* binding to a soluble form of the *IL-6* receptor (s*IL-6* R), enabling the *IL-6*/s*IL-6* R complex to activate gp130 on virtually all cell types, thereby expanding the spectrum of responsive cells within periodontal tissues. Both pathways converge on the activation of intracellular signaling cascades, particularly the Janus kinase/signal transducer and activator of transcription 3 (JAK/STAT3), mitogen-activated protein kinase (MAPK), and phosphoinositide-3-kinase/protein kinase B (PI3K/AKT) pathways. Through JAK/STAT3 activation, *IL-6* promotes the transcription of inflammatory cytokines and acute-phase proteins, perpetuating local immune responses. MAPK activation enhances the expression of adhesion molecules and pro-inflammatory mediators, facilitating leukocyte infiltration into gingival tissues. Meanwhile, PI3K/AKT signaling supports cellular survival and metabolic adaptation within the inflammatory microenvironment. Collectively, these pathways drive the expression of matrix metalloproteinases (MMPs), such as MMP-1, MMP-8, and MMP-9, which degrade extracellular matrix components and contribute directly to connective tissue breakdown and alveolar bone loss. Dysregulated *IL-6* signaling therefore represents a central mechanism linking the molecular inflammatory response to the clinical hallmarks of periodontitis, including pocket formation, attachment loss, and progressive tissue destruction [27].

In silico enrichment analysis further revealed that *IL-6* is significantly involved in NF-κB and IL-17 signaling pathways. Clinically, this corresponds to our findings that *IL-6* levels strongly correlated with PD and CAL, as NF-κB–mediated cytokine induction and IL-17–driven neutrophilic responses directly contribute to pocket formation, connective tissue degradation, and progressive bone loss. This alignment between pathway enrichment and clinical outcomes is further supported by Rodriguez-Montaño et al. [28], who demonstrated that IL-17A levels in gingival tissues are positively correlated with PD and CAL, and that IL-17 induces receptor activator of nuclear factor κB ligand (RANKL) expression and osteoclastogenesis, providing a mechanistic basis for the clinical observations [28].

Another important mechanism linking *IL-6* to periodontal tissue destruction is its regulation of bone metabolism. *IL-6* stimulates RANKL expression in osteoblasts and periodontal ligament fibroblasts, thereby enhancing the differentiation and activation of osteoclasts. This *IL-6* –RANKL–osteoclast axis accelerates alveolar bone resorption, which represents a key pathological feature of periodontitis. Inflammatory cells recruited to periodontal tissues further amplify this effect by releasing additional *IL-6*, establishing a feed-forward loop that sustains chronic bone loss. Thus, *IL-6* not only serves as an inflammatory mediator but also directly contributes to structural degradation by promoting osteoclastogenesis and alveolar bone destruction [29].

Beyond its local effects, *IL-6* may also have systemic consequences. Elevated *IL-6* generated in periodontal tissues can enter the systemic circulation, contributing to a heightened inflammatory burden. This spill-over effect is believed to underlie the well-documented associations between periodontitis and comorbid conditions such as cardiovascular disease, diabetes mellitus, and adverse pregnancy outcomes. Circulating *IL-6* stimulates hepatic production of C-reactive protein, a major risk marker for atherosclerosis, and has been implicated in insulin resistance through effects on adipose and hepatic metabolism. Therefore, elevated salivary *IL-6* levels may not only reflect periodontal status but also serve as a surrogate indicator of systemic inflammatory load, linking oral inflammation to broader systemic disease processes [30].

In addition to its direct effects, *IL-6* acts synergistically with other pro-inflammatory cytokines, particularly IL-1β and TNF-α, to perpetuate periodontal inflammation. IL-1β and TNF-α are rapidly induced in response to microbial biofilm and trigger the expression of adhesion molecules, chemokines, and prostaglandins within gingival tissues. *IL-6* sustains and amplifies these responses by promoting further cytokine secretion and acute-phase protein synthesis. Importantly, IL-1β and TNF-α also stimulate *IL-6* production, creating a self-reinforcing cytokine loop that maintains chronic inflammation. This synergistic interplay among *IL-6*, IL-1β, and TNF-α drives extracellular matrix degradation, osteoclastogenesis, and progressive alveolar bone resorption, thereby contributing to the irreversible tissue breakdown characteristic of advanced periodontitis [31].

Irisin, a myokine that has garnered increasing attention in recent years, has also been investigated in the context of periodontal diseases due to its roles in inflammation and bone metabolism [11]. Elevated irisin concentrations are thought to represent a physiological adaptation to heightened inflammatory activity [32,33]. Recent experimental data further support this concept: in a ligature-induced periodontitis rat model, gingival tissue irisin levels were significantly increased in the early phase of disease progression, and showed a negative correlation with TNF-α, while demonstrating positive associations with VEGF and EGF during periodontal repair [34]. These findings suggest that irisin may act as a dynamic marker of both tissue destruction and subsequent repair, reinforcing our observation of elevated salivary irisin levels in patients with Stage 3 Grade C periodontitis. In conditions characterized by acute inflammation or increased tissue stress, irisin released from muscle and other tissues is believed to rise in order to support tissue homeostasis [35]. In chronic diseases with episodic exacerbations, such as periodontitis, increased local tissue destruction and cellular stress may similarly stimulate irisin production. Indeed, recent studies have reported elevated irisin levels in individuals with high systemic inflammation and oxidative stress, suggesting that irisin may act as a protective and adaptive response [36,37]. Likewise, elevated irisin levels in individuals with periodontitis have been reported by Turkmen et al. and Khan et al. [12,13]. More recently, Khan et al. confirmed that salivary irisin levels were significantly higher in chronic periodontitis patients compared with healthy controls (6.80 vs. 3.99 ng/mL, *p* = 0.009), supporting its role as a potential non-invasive biomarker [13]. Consistent with these findings, our study demonstrated significantly higher irisin levels in the periodontitis group, with notable correlations observed between irisin and clinical parameters such as CAL, PD, and BOP. These results suggest that periodontitis is associated with systemic inflammatory burden, and that irisin may be linked to metabolic and inflammatory stress. However, contradictory findings regarding the association between these biomarkers and periodontitis have also been reported. For instance, Balci et al. found no significant difference in salivary *IL-6* levels between subjects with periodontitis and healthy controls [17]. Similarly, Anastasilakis et al. and Al Nimer noted that irisin levels do not consistently increase in systemic inflammatory conditions and may vary depending on factors such as disease stage, metabolic status, and sampling methodology [35,36]. In addition, Toraman et al. demonstrated that salivary IL-38 levels were significantly reduced in patients with periodontitis compared to healthy individuals, suggesting that not all cytokines are up-regulated during periodontal inflammation and that some, such as IL-38, may play an inhibitory or regulatory role in the disease process [38]. These conflicting findings underscore the complexity of the biological behavior of irisin and *IL-6* and their variability across different populations.

Ebersole et al. reported that *IL-6* exhibited sensitivity and specificity values ranging from 80% to 97%, with positive predictive values exceeding 90% [37]. Similarly, the ROC analysis results in our study are noteworthy: *IL-6* demonstrated exceptionally high diagnostic performance for periodontitis, with a sensitivity of 93.9% and a specificity of 97.0%, indicating near-perfect accuracy. Irisin also showed high diagnostic accuracy, with a sensitivity of 87.9%. In line with these findings, a recent systematic review and meta-analysis confirmed that the diagnostic accuracy of periodontitis is substantially improved when multiple salivary biomarkers, such as IL-1β, *IL-6*, and MMP-8, are evaluated in combination rather than individually [39]. The near-perfect AUC value observed for *IL-6* in our study may have been influenced by the relatively small sample size and the selection of distinct, extreme groups. Additionally, the use of a highly homogeneous and carefully selected study population might have contributed to this outcome. Therefore, further studies involving larger and more diverse populations are warranted to validate these findings.

A major strength of this study is its integrative approach, encompassing detailed clinical assessments alongside the concurrent measurement of two salivary biomarkers, thereby enhancing the reliability and depth of the findings. This study presents novel insights into the diagnostic and pathophysiological relevance of salivary *IL-6* and irisin levels in Stage 3 Grade C periodontitis. However, certain limitations must be acknowledged. First, the sample size, although statistically justified, was relatively limited and comprised well-defined and homogeneous groups, which may not fully represent the broader population affected by periodontitis. This controlled design, while enhancing internal validity, may have contributed to the near-perfect diagnostic performance of *IL-6* and irisin in ROC analysis and may not generalize to more diverse or intermediate clinical phenotypes. Second, the cross-sectional nature of the study prevents any causal inference regarding the role of these biomarkers in the initiation or progression of periodontal tissue destruction. Third, while in silico enrichment and molecular docking analyses provided mechanistic hypotheses, these computational predictions require further validation through in vitro and in vivo experimental studies to confirm biological interactions and functional implications. Finally, potential confounding factors such as unreported dietary habits, circadian variations in salivary biomarker secretion, or subclinical systemic inflammation were not accounted for and may have influenced biomarker concentrations. Recent evidence indicates that salivary analytes, including *IL-6*, exhibit measurable intra- and inter-day variability depending on daily activities such as eating or toothbrushing, yet remain reliable biomarkers for periodontitis detection, with *IL-6* showing the highest measurement stability [40]. These findings reinforce both the diagnostic value of *IL-6* and the need to consider pre-analytical conditions when interpreting salivary biomarker data. In addition, it is plausible that *IL-6* concentrations fluctuate between acute flare-ups and chronic stable phases of periodontitis, which may influence its temporal diagnostic utility and should be taken into account when interpreting salivary levels across different clinical contexts.

This study has several limitations that should be acknowledged. First, the relatively small sample size, although statistically justified, may limit the generalizability of the findings, as the study population consisted of carefully selected and homogeneous groups. Second, the cross-sectional design allows only the identification of associations rather than causation, and therefore no causal inferences can be made regarding the role of *IL-6* and irisin in the initiation or progression of periodontal tissue destruction. Third, while in silico enrichment and molecular docking analyses provided mechanistic insights, these computational predictions require further validation through experimental in vitro and in vivo studies. Additionally, potential confounding factors such as unreported dietary habits, circadian variations in salivary biomarker secretion, and subclinical systemic inflammation were not controlled for, and could have influenced biomarker levels. Finally, the near-perfect diagnostic performance observed in ROC analyses, particularly for *IL-6* (AUC = 0.99), is likely influenced by the relatively small and well-separated study groups. This may have led to an overestimation of diagnostic accuracy, and therefore the findings should be interpreted with caution until validated in larger and more heterogeneous populations.

From a translational standpoint, the pivotal role of *IL-6* in periodontal inflammation and tissue destruction also highlights it as a potential therapeutic target. Monoclonal antibodies that block *IL-6* signaling, such as anti-*IL-6* R agents (e.g., tocilizumab), have already demonstrated efficacy in systemic inflammatory disorders, including rheumatoid arthritis and juvenile idiopathic arthritis. Choy et al. reported that *IL-6* blockade with tocilizumab significantly reduced systemic inflammation and disease activity in rheumatoid arthritis, underscoring the feasibility of targeting this pathway in chronic inflammatory conditions [41]. While their application in periodontology has yet to be explored, inhibiting *IL-6* –mediated signaling could theoretically attenuate gingival inflammation, suppress osteoclastogenesis, and preserve periodontal tissue integrity. Future preclinical and clinical investigations are warranted to assess the safety and efficacy of *IL-6* -targeted therapies as adjuncts in periodontal disease management.

Future studies should aim to validate these findings in larger, multicenter cohorts with diverse demographic and clinical backgrounds. Longitudinal designs could help establish temporal relationships between biomarker fluctuations and disease progression or treatment response. Additionally, combining salivary *IL-6* and irisin with other emerging biomarkers and integrating machine learning-based diagnostic models may further enhance predictive accuracy and clinical applicability. Functional studies are also warranted to elucidate the molecular pathways by which *IL-6* and irisin may actively contribute to periodontal pathogenesis and systemic inflammatory crosstalk.

## 5. Conclusions

This study demonstrated that salivary irisin and *IL-6* concentrations were significantly elevated in individuals with Stage 3 Grade C periodontitis, aligning with clinical indicators of periodontal destruction such as PD, CAL, and BOP. Both biomarkers showed strong correlations with disease severity, and *IL-6*, in particular, exhibited excellent diagnostic performance with high sensitivity and specificity. Furthermore, in silico enrichment and molecular docking analyses revealed plausible biological pathways and a potential interaction interface between *IL-6* and irisin, suggesting their possible association with periodontal inflammation and tissue metabolism. These findings support the utility of salivary *IL-6* and irisin not only as non-invasive diagnostic biomarkers but also as potential contributors to the pathophysiology of periodontitis. Nonetheless, larger-scale and longitudinal studies are warranted to confirm these results and to further explore their mechanistic roles and translational relevance.

## Figures and Tables

**Table 1:** Comparison of demographic, clinical, and biochemical parameters between healthy and periodontitis groups.

Parameter	Healthy (C, *n* = 33)	Periodontitis (P, *n* = 33)	Test	*p*-Value
Age (years)	28.73 ± 5.20	45.70 ± 6.77	Mann–Whitney U	*p* < 0.001 *
Sex (Male/Female)	15/18	16/17	Chi-square	0.82 (n.s.)
BMI (kg/m^2^)	23.34 ± 1.10	23.46 ± 1.13	*t*-test	0.685
Educational background	55% Univ./45% HS	52% Univ./48% HS	Chi-square	0.79 (n.s.)
Oral hygiene (regular brushing ≥2/day)	58%	55%	Chi-square	0.77 (n.s.)
PD (mm)	1.77 ± 0.36	5.02 ± 0.40	*t*-test	*p* < 0.001 *
BOP (%)	7.08 ± 1.27	42.32 ± 6.25	Mann–Whitney U	*p* < 0.001 *
CAL (mm)	0.00 ± 0.00	5.58 ± 0.56	Mann–Whitney U	*p* < 0.001 *
PI	0.58 ± 0.13	2.08 ± 0.24	*t*-test	*p* < 0.001 *
*IL-6* (ng/L)	25.07 ± 5.28	71.76 ± 7.54	Mann–Whitney U	*p* < 0.001 *
Irisin (ng/mL)	9.94 ± 3.09	28.85 ± 7.99	*t*-test	*p* < 0.001 *

* Data are presented as mean ± standard deviation (SD) or *n* (%). HS: High School; Univ.: University; PD: Probing Depth; CAL: Clinical Attachment Loss; BOP: Bleeding on Probing; PI: Plaque Index. Percentages for sex, education, and oral hygiene represent approximate averages based on national-level data for Turkey. *p* < 0.05 was considered statistically significant. n.s.: not significant.

**Table 2:** Correlation analysis of biomarkers with clinical and demographic parameters.

Group	Variable	Biomarker	Spearman Rho	*p*-Value
Control (C)	BMI	*IL-6* (ng/L)	0.429	0.013 *
Periodontitis (P)	PI	*IL-6* (ng/L)	0.403	0.020 *
All groups (C + P)	Age	*IL-6* (ng/L)	0.673	*p* < 0.001 *
All groups (C + P)	Age	Irisin (ng/mL)	0.683	*p* < 0.001 *
All groups (C + P)	PI	*IL-6* (ng/L)	0.821	*p* < 0.001 *
All groups (C + P)	PI	Irisin (ng/mL)	0.728	*p* < 0.001 *
All groups (C + P)	PD	*IL-6* (ng/L)	0.739	*p* < 0.001 *
All groups (C + P)	PD	Irisin (ng/mL)	0.726	*p* < 0.001 *
All groups (C + P)	BOP	*IL-6* (ng/L)	0.772	*p* < 0.001 *
All groups (C + P)	BOP	Irisin (ng/mL)	0.722	*p* < 0.001 *
All groups (C + P)	CAL	*IL-6* (ng/L)	0.803	*p* < 0.001 *
All groups (C + P)	CAL	Irisin (ng/mL)	0.693	*p* < 0.001 *

Spearman correlation test. Only correlations with * *p* < 0.05 are presented. PI, Plaque Index; BOP, Bleeding on Probing; PD, Probing Depth; CAL, Clinical Attachment Loss; BMI, Body Mass Index.

**Table 3:** Diagnostic performance of *IL-6* and irisin for distinguishing periodontitis according to Receiver operating characteristic (ROC) analysis.

Marker	AUC	Cut-Off	Sensitivity (%)	Specificity (%)
*IL-6*	0.99	37.87	93.9	97.0
Irisin	0.95	14.91	87.9	100.0

## Data Availability

The data supporting this study’s findings are available from the corresponding authors upon reasonable request.
